# Notch1 signaling in melanoma cells promoted tumor-induced immunosuppression via upregulation of TGF-β1

**DOI:** 10.1186/s13046-017-0664-4

**Published:** 2018-01-04

**Authors:** Zike Yang, Yanxia Qi, Nan Lai, Jiahe Zhang, Zehong Chen, Mingyu Liu, Wan Zhang, Rongcheng Luo, Shijun Kang

**Affiliations:** 1 Cancer Center, Integrated Hospital of Traditional Chinese Medicine, Southern Medical University, No.13, Shiliugang Road, Haizhu District, Guangzhou, 510315 Guangdong Province People’s Republic of China; 2 Oncology Department, Nanfang Hospital, Southern Medical University, No.1838, North of Guangzhou Avenue, Baiyun District, Guangzhou, Guangdong Province 510515 People’s Republic of China; 3 Cancer Center, The First People’s Hospital of Huaihua City, Huaihua, 418000 Hunan Province People’s Republic of China

**Keywords:** Malignant melanoma, Immunotherapy, Immunosuppression, Notch1, TGF-β1, Notch

## Abstract

**Background:**

The receptors of Notch family play an important role in controlling the development, differentiation, and function of multiple cell types. The aim of this study is to investigate the role of Notch1 signaling upon immune suppression induced by melanoma cells.

**Methods:**

Melanoma cell line B16 cells were transfected by lentivirus containing mouse Notch1 gene or Notch1 shRNA to generate B16 cell line that highly or lowly expressed Notch1. Notch1 in anti-tumor immune response was comprehensively appraised in murine B16 melanoma tumor model in immunocompetent and immunodeficient mice. The ratios of CD3^+^CD8^+^ cytotoxic T cells, CD49b^+^NK cells, CD4^+^CD25^+^FoxP3^+^ Tregs and Gr1^+^CD11b^+^ MDSCs in tumor-DLN or spleen were examined by flow cytometry. After the co-culture of B16 cells and CD8^+^ T cells, the effects of Notch1 on the proliferation and activation of T cells were assessed by CCK8 assay, CFSE dilution and Chromium-release test. The mRNA expression and supernatant secretion of immunosuppressive cytokines, TGF-β1, VEGF, IL-10 and IFN-γ were measured by RT-PCR and ELISA, respectively.

**Results:**

Downregulation or overexpression of Notch1 in B16 melanoma cells inhibited or promoted tumor growth in immunocompetent mice, respectively. Notch1 expression in B16 melanoma cells inhibited the infiltration of CD8+ cytotoxic T lymphocytes and NK cells and reduced IFN-γ release in tumor tissue. It could also enhance B16 cell-mediated inhibition of T cell proliferation and activation, and upregulate PD-1 expression on CD4^+^ and CD8^+^ T cells. The percentage of CD4^+^CD25^+^FoxP3^+^ Tregs and Gr1^+^CD11b^+^MDSCs were significantly increased in tumor microenvironment, and all these were attributed to the upregulation of TGF-β1.

**Conclusion:**

These findings suggested that Notch1 signaling in B16 melanoma cells might inhibit antitumor immunity by upregulation of TGF-β1.

## Background

Malignant melanoma, one of the most highly aggressive tumors, resists to conventional chemotherapy and radiotherapy and has fatal outcomes. There are compelling evidences to show that melanoma cells escape the host’s immunity by actively developing multiple suppressive mechanisms within the cancer microenvironment [1]. For instance, melanoma cells evade T cell surveillance by creating an immunosuppressive environment via the production of cytokines such as transforming growth factor (TGF)-β1, vascular endothelial growth factor (VEGF) and IL-10, which recruit myeloid-derived suppressor cells (MDSCs) and T regulatory cells (Tregs). The promotion and recruition of MDSCs and Tregs by melanoma cells play a crucial role in tumor immune escape [2].

The Notch signaling is a highly conserved pathway that controls the differentiation, development and function of multiple cell types, such as stem cells [3]. Mammals have four Notch receptors (Notch1, Notch2, Notch3, and Notch4) that are bound by five ligands (Jagged-1, Jagged-2, DLL1, DLL3, and DLL4) families [4]. Aberrant Notch signaling has been identified in malignant melanoma to play an important role in the malignant biological behavior of melanoma [5]. Our previous study has shown that interference of both Notch co-activation factor MAML1 blocks the activation of Notch pathway in both human and mouse melanoma cells, suggesting a potential new treatment strategy [6]. Among the 4 receptors, Notch2-4 have been identified in multiple cell types, such as stem cells, hematopoietic cells, macrophage or nerve cells, and controlled their differentiation, development and function [7, 8]. The role of Notch1 has been proved to be closely related to melanoma progression and become a research hotspot recently [9]. Previous studies have demonstrated that Notch1 signaling promoted primary melanoma progression by activating mitogen-activated protein kinase/phosphatidylinositol 3-kinase-Akt pathways and up-regulating N-cadherin expression [10]. Moreover, Notch1 and NRG1 expression in melanoma promoted cell growth by activating PI3Kinase/Akt signaling pathway and facilitating the accumulation of p27 [11]. Additionally, activated Notch1 receptors in endothelial cells promoted neutrophil infiltration, tumor cell adhesion to the endothelium, intravasation, lung colonization and facilitated melanoma metastasis by generating a senescent, pro-inflammatory endothelium [12].

Although Notch signaling is known to be important for the malignant biological behavior of melanoma cells, little is known about the effects of aberrant activation of this pathway in melanoma on tumor-induced immunosuppressive microenvironment. Our primary study has shown that siRNA-mediated Notch1 knockdown might potentially enhance the effect of IL-2 immunotherapy in malignant melanoma [13]. In the present study, we further evaluated the role of Notch1 expression in melanoma cells on tumor-induced immunosuppression. This study was not only important for elucidating the mechanism of tumor-induced immune escape, but also provided a scientific basis for developing novel immunotherapeutic strategies to target Notch1 in B16 melanoma cells to induce innate and adaptive immune responses against tumors.

## Methods

### Cells and animals

Murine malignant melanoma cell line B16 was purchased from China Center for Type Culture Collection. B16 cells were cultured in DMEM-high glucose (Thermo Fisher Scientific, Waltham, MA, USA) supplemented with 10% fetal bovine serum (Thermo Fisher Scientific, Waltham, MA, USA) at 37 °C in an atmosphere of 5% CO^2^.

### In vivo study

Female C57BL/6 and BALB/c Nude mice were purchased from Laboratory Animal Center of Southern Medical University (Guangzhou, China). All mice were 6- to 8 weeks of age at the time of experiment, and at least 6 mice per group were used in each experiment. For tumor challenge experiments, 5 × 10^5^ B16, B16-shNotch1 or B16-Notch1 melanoma cells were subcutaneously inoculated. Mice were observed carefully while tumor volume in mice was measured twice a week. Tumor volume was calculated by (length × width^2^)/2. All mice were sacrificed humanely after the experiments. Melanoma tissues in mice were surgically resected to perform further assay. Animal care and treatment were in accordance with institutional guidelines. All animal study protocols were reviewed and approved by the Animal Care and Use Committee of Southern Medical University, China.

### Generation of stable Notch1 overexpression and knockdown melanoma cells

Lentivirus containing mouse Notch1 (lentivirus-mNotch1) and Notch1 shRNA (lentivirus-shNotch1) were constructed by gene technology company (Genechem, Shanghai, China). The day before infection, cells were plated at a density of 20~30% in a good condition. The lentivirus above (containing fluorescence) were used to transfect B16 cells according to the manufacture’s protocol. The culture medium was changed to normal medium 12 h after infection. Three days later, the cells were subjected to fluorescence activated cell sorting (FACS) with flow cytometer for further experiments.

### ELISpot assays

Mouse IFN-γ ELISpot kits (BD Biosciences, USA) were used according to the manufacturer’s instructions. Briefly, immune cells from spleens or tumor-DLNs (1 × 10^5^) were co-cultured with 30 Gy-irradiated B16 (5 × 10^4^) cells in 96-well plates precoated with mouse IFN**-γ** for 20 h at 37 °C in complete DMEM medium in triplicate. The cell suspension was aspirated and washed with deionized water. Then Biotinylated anti-mouse IFN**-γ** antibody was added and incubated for 2 h at room temperature. After washing with deionized water, a streptavidin-horseradish peroxidase solution was added and incubated for 1 h at room temperature. Then an aminoethyl carbozole substrate solution was added and incubated for 15 min. Spots in plate were counted using a stereomicroscope after washing.

### Cell surface marker and intracellular cytokine staining

At the indicated time points, tumor draining lymph nodes(tumor-DLNs) and spleens were harvested from the mice, and minced into small fragments and mechanically dispersed in 3-5 ml cold PBS. After filtering with 70 μm cell strainer (BD Falcon, USA), Single-cell suspensions were adjusted to 1 × 10^6^ cells in 100 μl of PBS. After this, single-cell suspensions of tumor cells were stained for 30 min on ice with 1 μg of antibodies labeled with fluorochromes CD45, CD3, CD4, CD8, CD49b, CD25, CD11b, Gr-1 or matched isotypic control antibodies(BD Biosciences, USA) and then fixed and permeabilized with a permeabilization buffer (BD Biosciences, USA). Cells were finally stained with antibody to IFN**-γ** or FoxP3 for 50 min at 4 °C, washed again, and analyzed by FACSCalibur (BD Biosciences, USA). Irrelevant IgG mAbs were used as a negative control. Ten thousands live events were acquired for analysis.

### In vivo depletion of T and NK cells

To deplete CD8^+^T, CD4^+^T and NK cells before and during treatment with lentivirus-shNotch1, the transplanted mice received intravenous injections of 0.3 mg mAb from anti-CD8^+^ hybridoma (Bioxcell, USA) the anti-CD4^+^ hybridoma (Bioxcell, USA) or 0.5 mg of antiasialo-GM1 antibody (Wako Pure Chemical Industries, Ltd). Antibody injection was started on the day of tumor innoculation, and the treatment was repeated every 5 days throughout the entire experimental period to ensure the depletion of the target immune cell subset.

### Splenic T lymphocyte isolation

Splenic lymphocytes were isolated from splenic cell suspension using density gradient centrifugation (Ficoll-Hypaque, TBD Science, Tianjin, China). CD8^+^ T lymphocytes were purified using microbead isolation kits followed by magnetic-activated cell sorting (MACS) according to the manufacturer’s instructions (Miltenyi Biotec, Germany). Isolated cells were resuspended at a concentration of 1 × 10^6^ cells/mL in RPMI-1640 medium (Thermo Fisher Scientific, USA) containing L-glutamine (2 mM), penicillin/streptomycin (100 U/mL), and 10% fetal calf serum (Thermo Fisher Scientific, Waltham, MA, USA).

### Lymphocyte proliferation assay and apoptosis analysis

3.0 μm Transwell chamber(Corning, USA) were used for co-culture of CD8^+^T cells and B16 cells. B16 cells were plated in lower chamber with 10^4^ cells per well. CD8^+^T lymphocytes were then added to the upper chamber in 1:1 ratio. For CFSE analysis, CD8^+^T cells were labelled with CFSE staining before co-cultured with B16 cells. After co-cultured for 48 h, CD8^+^ cells in upper chamber were harvested for CCK8 assay or analysis of CFSE dilution and apoptosis.

### In vitro cytotoxicity assay

CD8^+^ T cells isolated from B16 tumor-bearing mice were cultured for 3 days with irradiated B16 stimulators in a complete 1640 RPMI medium. The responder cells were then collected and used as effector cells in a 8 h chromium release assay against indicated B16 target cells. B16 target cells were labeled by combining 5 × 10^6^ cells with 50 μCi 51Cr (Perkin-Elmer Japan Co.) in a total volume of 0.2 ml complete RPMI for 1 h at 37 °C, followed by washing thrice with plain RPMI. For the chromium release assay, CD8^+^ effector cells were mixed with B16 target cells at the different ratio of 1:1, 5:1 and 25:1 in a 96-well round-bottom plate (BD Biosciences).

### Enzyme-linked immunosorbent assay (ELISA)

B16 cells were seeded into 6-well plates at a density of 2 × 10^5^ cells/ml. The culture supernatants were harvested after B16 cells had adhered to the bottom of the culture plate for 18h. Then, the supernatants were centrifuged to remove melanoma cells. In accordance with the manufacturer’s instructions, Culture supernatants of B16 cells were measured by ELISA for the secretion of TGF-β1, VEGF and IL-10 (eBioscience, San Diego, CA, USA). Freshly excised tumor tissues were minced and homogenized for 1 min in tissue tearor. Then, supernatants were collected after centrifugation and subjected to ELISA for examining the secretion of TGF-β1, VEGF, IL-10 and IFN-γ using mouse ELISA kits according to the manufacturer’s protocol (eBioscience, San Diego, CA, USA).

### Statistical analysis

All data were expressed as means ±SEM. Results of tumor volume, flow cytometry, Western blotting, ELISA, inhibition rate on lymphocyte proliferation and in vitro cytotoxicity were assessed using a student’s t-test. Differences were considered to be statistically significant when *P* < 0.05. All statistical analyses were performed using SPSS 20.0 (IBM, Armonk, NY, USA).

## Results

### Notch1 expression in melanoma cells promoted tumor growth in vivo

**Fig. 1:**
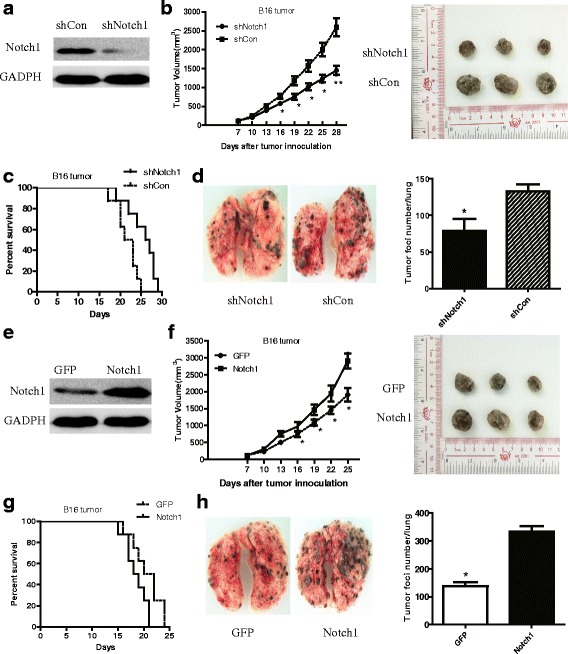
Notch1 expression in melanoma cells promoted tumor growth in vivo. **a** Western blot analysis of Notch1 expression in shNotch1 and shCon cells. **b** Tumor growth curve of shNotch1 and shCon cells in C57 BL/6 mice. The representative three subcutaneous tumor were shown. **c** Overall survival rates of shNotch1 and shCon cells in C57 BL/6 mice. **d** The number of lung tumor foci in C57BL/6 mice intravenously inoculated with B16-shNotch1 or B16-shCon cells. The representative one lung tumor was shown. **e** Western blot analysis of Notch1 expression in Notch1 overexpressing and GFP control cells. **f** Tumor growth curve of Notch1 and GFP cells in C57 BL/6 mice. The representative three subcutaneous tumor were shown. **g** Overall survival rates of GFP and Notch1 cells in C57 BL/6 mice. **h** The number of lung tumor foci in C57BL/6 mice intravenously inoculated with B16-Notch1 or B16-GFP cells. The representative one lung tumor was shown. 8 mice per group were used in this experiment. *, *P* < 0.05. “ns”, no statistical significance

To examine the role of Notch1 in tumor growth, we stably silenced Notch1 expression using shRNA in B16 tumor cells (Fig. 1a). B16-shNotch1(Notch1 knockdown) or B16-shCon(control) cells were subcutaneously inoculated into immunocompetent C57BL/6 mice. Knocking down Notch1 significantly retarded tumor growth in C57BL/6 mice and extended mice survival time (Fig. 1b & c). To further confirm the effect of Notch1 on tumor growth, we generated a B16 cell line that highly expressed Notch1 (B16-Notch1) by lentivirus expression vector (Fig. 1e). When B16 cells transfected with a control vector (B16-GFP) and B16-Notch1 cells were inoculated into immunocompetent C57BL/6 mice, overexpression of Notch1 significantly increased tumor burden and shortened mice survival time (Fig. 1f & g).

In order to confirm this finding, we intravenously inoculated B16-shCon, B16- shNotch1, or B16-Notch1 melanoma cells into C57BL/6 mice. The number of lung tumor foci in C57BL/6 mice was counted on day 14 after intravenous inoculation. Pulmonary metastasis in mice receiving B16-shNotch1 cells was significantly inhibited, whereas pulmonary metastasis was enhanced in those receiving B16-Notch1 melanoma cells (Fig. 1d & h).

### Notch1 function in tumor growth depends on the immune system

**Fig. 2:**
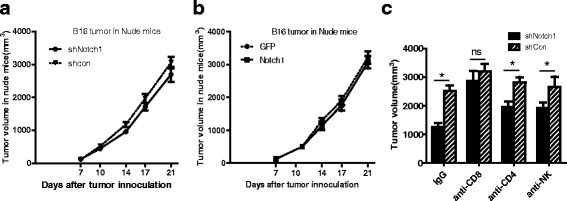
Notch1 function in tumor growth depended on the immune system. **a** Tumor growth curve of shNotch1 and shCon cells in BALB/c Nude mice. **b** Tumor growth curve of Notch1 and GFP cells in BALB/c Nude mice. **c** Tumor volume of shNotch1 and shCon cells on day 28 in C57 BL/6 mice with or without depletion of CD8^+^ or CD4^+^ T cells, or NK cells. 8 mice per group were used in this experiment. *, *P* < 0.05. “ns”, no statistical significance

To explore whether regulation of tumor growth by Notch1 expression in melanoma cells was due to host immune status, we subcutaneously inoculated B16- shCon, B16-shNotch1, or B16-Notch1 melanoma cells into BALB/c Nude mice, which are deficient in T, B and NK cells. In contrast to immunocompetent mice, no significant differences of tumor burden were observed in BALB/c Nude mice challenged with B16-shNotch1 or B16-Notch1 cells (Fig. 2a & b). The results suggested that Notch1 might promote tumor growth in vivo via suppression of antitumor immunity.

To further determine how Notch1 affected the antitumor immunity, B16-shNotch1 or B16-shCon cells were subcutaneously inoculated into C57BL/6 mice that were depleted of CD4^+^ or CD8^+^ T cells or NK cells and tumor growth was monitored. CD8^+^ T cell depletion significantly compromised the effect of Notch1 expression on tumor growth. However, the compromised effect of CD4^+^ T cells or NK cells depletion was less important than CD8^+^ T cells in Notch1-mediated tumor growth (Fig. 2c). These results showed that regulation of tumor growth by Notch1 majorly depended on CD8^+^ T cells in immune system.

### Notch1 inhibited local antitumor immunity but not system immunity

**Fig. 3:**
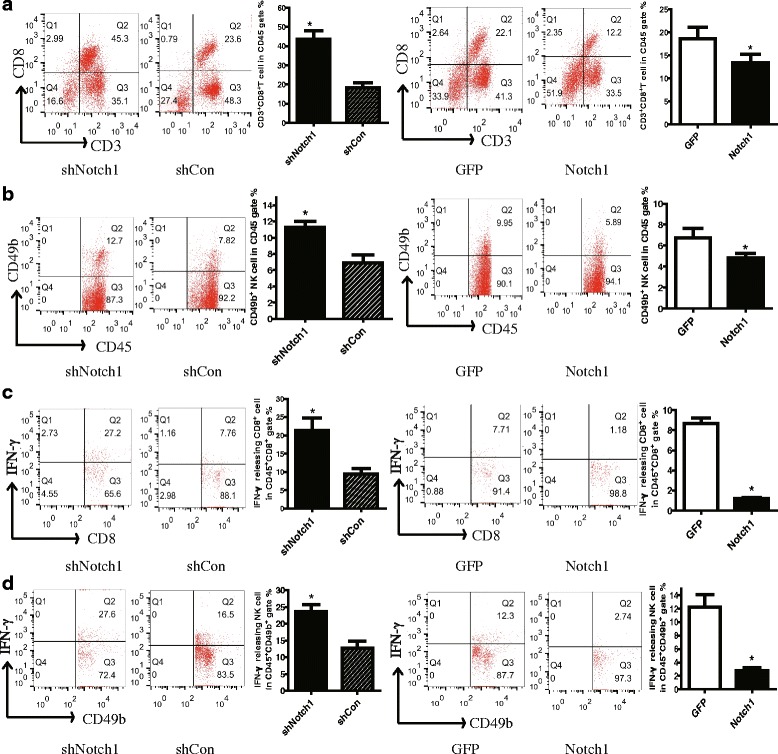
Notch1 expression in melanoma cells inhibited IFN-γ-releasing CTL and NK cell infiltration in tumor . Tumor-DLNs isolated from tumor-bearing mice were analyzed for CD3^+^ CD8^+^ T cells, CD49b^+^ NK cells and IFN-γ-releasing immune cells by flow cytometry. **a** Flow cytometry analysis of CD3^+^ CD8^+^ T lymphocytes in CD45^+^ gate in tumor-DLNs. **b** Flow cytometry analysis of CD49b^+^ NK cells in CD45^+^ gate in tumor-DLNs. **c** Flow cytometry analysis of IFN-γ-releasing CTL in CD45^+^ gate in tumor-DLNs. **d** Flow cytometry analysis of IFN-γ-releasing CD49b^+^ NK cells in CD45^+^ gate in tumor-DLNs. Data was pooled from 3 independent experiments. *, *P* < 0.05

To investigate the effect of Notch1 in tumor tissue on local anti-tumor immunity, we analyzed the immune status in the tumor microenvironment of B16 tumor-bearing mice. We found that knocking down Notch1 remarkably reduced while overexpressing Notch1 increased the percentage of CD3^+^CD8^+^ CTL and NK cells in tumor-DLNs (Fig. 3a & b). As IFN-γ is a cytokine crucial for T-cell activation and effector function, we then examined the IFN-γ-expressing immune cells in tumor-DLNs. Knocking down Notch1 resulted in a significant increase of the percentage of IFN-γ-expressing CD8^+^ T cells and IFN-γ-expressing NK cells (Fig. 3c & d). In contrast, the opposite results were observed in Notch1 overexpressing cells (Fig. 3a-d). These results suggested that Notch1 in tumor cells remarkably inhibited local antitumor immunity.

**Fig. 4:**
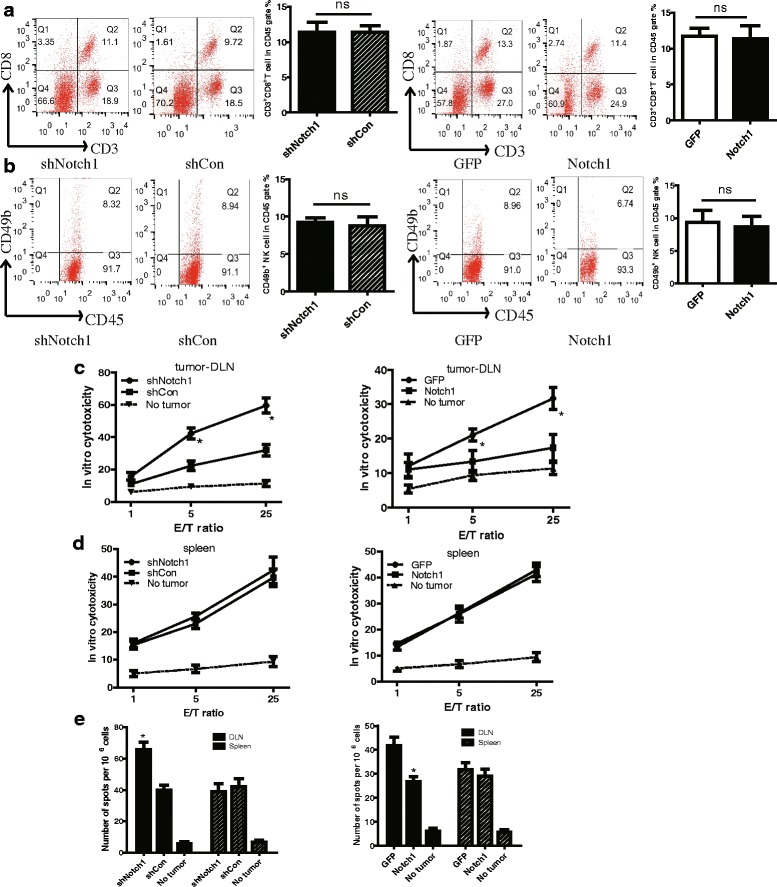
Notch1 in tumor tissues inhibited CTL activation in tumor-DLNs but not in spleen cells. Splenocytes isolated from tumor-bearing mice were analyzed for CD3^+^ CD8^+^ T cells and CD49b^+^ NK cells by flow cytometry. **a** Flow cytometry analysis of CD3^+^ CD8^+^ T lymphocytes in CD45^+^ gate in spleens. **b** Flow cytometry analysis of CD49b^+^ NK cells in CD45^+^ gate in spleens. **c** In vitro cytotoxicity of CD8^+^ tumor-DLNs cells from B16 tumor-bearing mice against B16 cells in co-culture system. CD8^+^ T effector cells purified by positive selection from tumor-DLNs of in C57BL/6 mice subcutaneously inoculated with Notch1 knockdown or overexpressing cells were co-cultured with wild-type B16 cells at the different ratio of 1:1, 5:1, and 25:1. Tumor-DLNs isolated from non-tumor bearing mice were used as a baseline control. **d** In vitro cytotoxicity of CD8^+^ splenocytes from B16 tumor-bearing mice against B16 cells in co-culture system. Spleens isolated from non-tumor bearing mice were used as a baseline control. **e** IFN-γ ELISPOT assay was performed in splenocytes and tumor-DLN cells isolated from B16 tumor-bearing mice and restimulated with irradiated B16. Data was pooled from 3 independent experiments. *, *P* < 0.05. "ns", no statistical significance

We next analyzed the spleen T lymphocytes to evaluate the system immunity. Knocking down or overexpressing Notch1 did not affect the percentage of CD3^+^CD8^+^ CTL and NK cells in splenocytes (Fig. 4a & b). Then, IFN-γ ELISPOT assay and chromium release assay were performed to evaluate the cytotoxicity of T cells in tumor-DLNs and spleens. In in vitro cytotoxicity assay, knocking down Notch1 significantly increased the CTL activity in tumor-DLNs but not in spleens (Fig. 4c & d). Consistent with the chromium release assay, we found that knocking down Notch1strongly increased the number of IFN-γ–producing cells in tumor-DLNs in response to B16 tumor antigen (Fig. 4e). In contrast, the opposite results were observed in Notch1 overexpressing cells. These data suggested that Notch1 in tumor tissues inhibited CTL activation in tumor-DLNs but not in spleen cells.

### Notch1 expression in tumor modulated immune cell population and phenotype

**Fig. 5:**
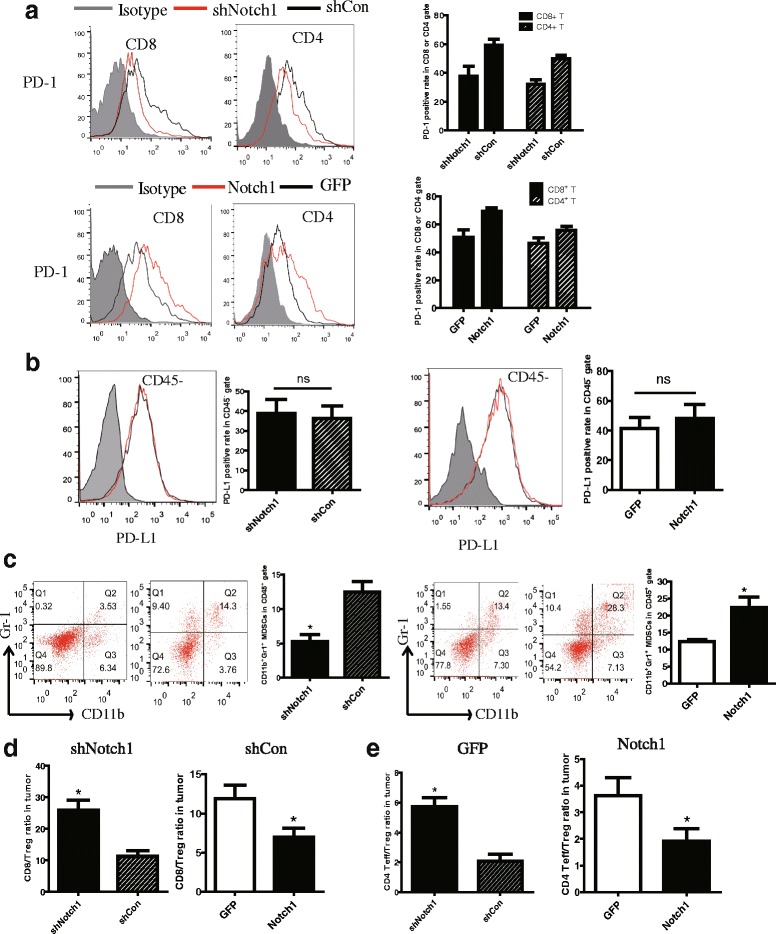
Notch1 expression in tumor modulated immune cell population and phenotype. Tumor-DLNs and tumor tissue isolated from tumor-bearing mice were analyzed for immune cell population and phenotype by flow cytometry. **a** Flow cytometry analysis of PD-1 expression on CD4^+^ and CD8^+^ T lymphocytes in tumor-DLNs. **b** Flow cytometry analysis of PD-L1 expression on CD45^−^ melanoma cells in tumor. **c** Flow cytometry analysis of CD11b^+^Gr-1^+^ MDSCs on CD45^+^ gate in tumor-DLNs. **d** CD8^+^ T cells to Tregs ratios in tumor-DLNs. **e** CD4 ^+^ Effector cells to Tregs ratios in tumor-DLNs. Data was pooled from 3 independent experiments. *, *P* < 0.05

The PD-1/PD-L1 axis is a critical regulator of T cell fate and function. PD-1 has also been identified as a marker of T cell exhaustion, a hypo-functional cell state found in tumor. The expression of PD-1 on CD8^+^ and CD4^+^ T cells in tumor-DLNs were analyzed by flow cytometry. Knocking down Notch1 reduced PD-1 expression while overexpressing Notch1 showed an increased PD-1 expression on CD8^+^ and CD4^+^ T cells (Fig. 5a). No significant difference was observed in PD-L1 expression on CD45^−^ melanoma cells in tumor (Fig. 5b). Tregs and MDSCs, as the immunosuppressor cells, suppress antitumor immunity and weaken therapeutic efficacy of immunotherapy. To investigate the mechanism how CTLs were inhibited through Notch1 in the tumor microenvironment, we assessed the percentage of MDSCs and Tregs in tumor-DLNs of either Notch1 knocked down or knocked in cells. As shown in Fig. 5c-e, Notch1 in melanoma tumor promoted more MDSCs and Tregs infiltration in tumor microenvironment and suppressed antitumor immunity (Fig. 5c-e).

### Notch1 enhanced the inhibitory effects of melanoma cells on proliferation and activation of CD8^+^T lymphocytes

**Fig. 6:**
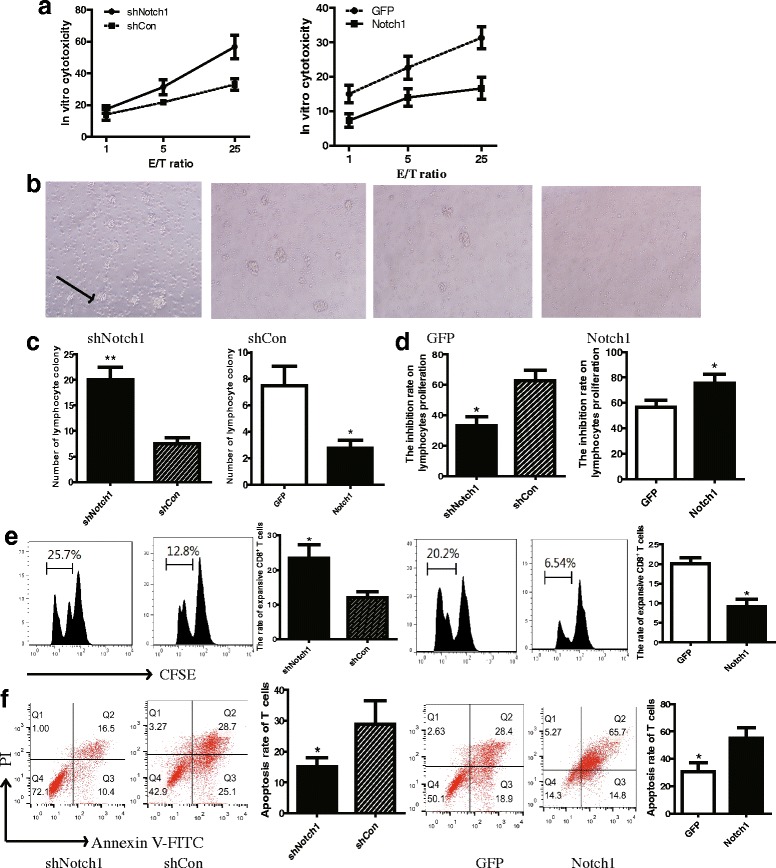
Notch1 expression in melanoma cells increased tumor cell-mediated inhibition of T cell proliferation and activation. **a** In vitro cytotoxicity of CD8^+^ T lymphocytes against Notch1 knockdown or overexpressing cells. CD8^+^ T lymphocytes isolated from wild-type B16 (unmodified) tumor-bearing mice were activated by CD3 antibody, CD28 antibody, and irradiated B16 cells for 3 days. Then the CD8^+^ effector cell were co-cultured with Notch1 knockdown or overexpressing cells at the different ratio of 1:1, 5:1, and 25:1. In vitro cytotoxicity of CD8^+^ T lymphocytes against Notch1 knockdown or overexpressing cells was detected by chromium release assay. **b** Lymphocyte colony formation of CD8^+^ T cell after co-cultured with B16 cells (magnification, 200×). Black arrows indicated lymphocyte colony formation. Total CD8^+^ T cells purified by positive selection from spleens of wild-type B16 tumor-bearing mice were co-cultured with Notch1 knockdown or overexpressing cells at the ratio of 1:1 in transwell chamber (Con A 5 μg/ml). **c** Number of lymphocyte colony. **d** The inhibition rate of lymphocyte proliferation by B16 cells was detected by CCK8 kit. **e** CD8^+^ T lymphocytes labeled with CFSE were analyzed for proliferation by flow cytometry. **f** The apoptosis of CD8^+^ T lymphocytes were analyzed by flow cytometry after 7 days. Results shown were representative of 3 independent experiments. *, *P* < 0.05. **, *P* < 0.01

To explore the mechanism of immunosuppression induced by Notch1 in B16 cells, splenic CD8^+^ T cells isolated from B16 tumor-bearing mice were expanded and activated with irradiated B16 cells in culture for 3 days prior to test cytotoxic function against B16-shNotch1 or B16-Notch1 cells by chromium release assays. The cytotoxicity of T cells against B16-shNotch1 was more powerful than that against B16-shCon cells (Fig. 6a). The diminished effects were observed in Notch1 overexpressing cells (Fig. 6a).

To evaluate the role of Notch1 in B16 cells on inhibiting T lymphocyte proliferation, CD8^+^T cells isolated from wild-type B16 tumor-bearing mice were co-cultured with Notch1 knocked down or knocked in B16 cells in transwell chamber for 48 h. The CD8^+^T cells were cultured in the upper chamber and B16 cells were in the lower chamber. According to microscopic data, knocking down Notch1 showed more lymphocyte colony formation than control group (Fig. 6b & c). In CCK8 lymphocyte proliferation assay, the proliferation rate was significantly increased in CD8^+^T cells which were co-cultured with B16-shNotch1 cells (Fig. 6d). The effects of Notch1 on inhibiting T lymphocytes proliferation was confirmed by CFSE dilution (Fig. 6e). Moreover, less T cells were induced apoptosis after co-cultured with B16-shNotch1 cells (Fig. 6f). In contrast, the opposite results were observed in Notch1 overexpressing cells (Fig. 6b-f). These results suggested that Notch1 promoted the inhibitory effects of melanoma cells on lymphocyte proliferation and activation.

### The effects of Notch1 on tumor-induced immunosuppression were attributed to the upregulation of TGF-β1

**Fig. 7:**
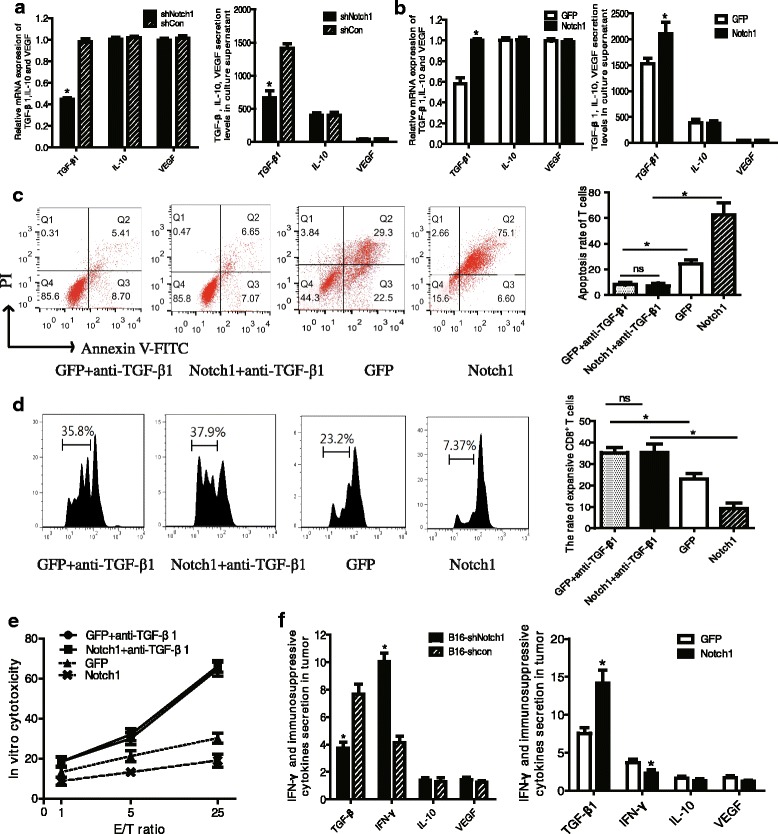
The effects of Notch1 on tumor-induced immunosuppression were attributed to the upregulation of TGF-β1. **a** Relative mRNA expression of TGF-β1, IL-10 and VEGF in Notch1 knockdown or overexpressing B16 cells. **b** TGF-β1, IL-10 and VEGF secretion levels in culture supernatant of Notch1 knockdown or overexpressing B16 cells were determined by ELISA. **c** The apoptosis of CD3^+^ T lymphocytes were analyzed in TGF-β1 neutralization test. **d** CD3^+^ T lymphocytes labeled with CFSE were analyzed for proliferation after anti-TGF-β1 administration. **e** In vitro cytotoxicity of CD8^+^ T lymphocytes against Notch1 knockdown or overexpressing cells after TGF-β1 was neutralized. **f** Secretions of IFN-γ and immunosuppressive cytokines TGF-β1, VEGF and IL-10 in homogenate supernatant of tumor tissues were determined using ELISA. 8 mice per group were used in this experiment. *, *P* < 0.05

To study how Notch1 affect local anti-tumor immunity in vivo and vitro, mRNA expression and supernatant secretions of TGF-β1, VEGF and IL-10 in B16 cells were measured by Q-PCR and ELISA, respectively. Notch1 knocking down remarkably reduced, while overexpression of Notch1 showed significant increase in mRNA expression and supernatant secretion of TGF-β1 (Fig. 7a & b). However, no significant differences were observed in mRNA expression and supernatant secretion of IL-10 and VEGF (Fig. 7a & b). The results indicated that Notch1 upregulated tumor-derived TGF-β1 in melanoma cells. In addition, we discovered that neutralization of TGF-β1 in the co-culture system significantly reduced the inhibitory effects of Notch1 on CD8^+^T lymphocyte apoptosis (Fig. 7c), proliferation (Fig. 7d) and activation (Fig. 7e) induced by melanoma cells.

To determine the secretion of immunosuppressive cytokines and IFN-γ in vivo, homogenate of tumor tissue from B16 tumor-bearing mice were harvested for ELISA assay. The data showed that TGF-β1 secretion in tumor tissue was significantly reduced in B16-shNotch1 tumor, while increased in B16-Notch1 tumor in vivo (Fig. 7f). However, there was no significant change in IL-10 and VEGF secretions in all groups of mice (Fig. 7f). In addition, IFN-γ secretion of homogenate supernatant in B16-shNotch1 tumor was significantly increased, suggesting an enhancement of antitumor immunity (Fig. 7f). In contrast, Notch1 overexpressing tumor showed a reduction of IFN-γ secretion in homogenate supernatant. These data indicated that Notch1 in melanoma up-regulated melanoma-derived TGF-β1 and promoted tumor-induced immunosuppression in vivo.

## Discussion

Malignant melanoma is a potentially fatal cancer characterized by rapid progression, metastasis to regional lymph nodes and distant organs, and resistance to chemotherapy and radiotherapy [14]. Melanoma has well-documented immunogenicity, which is conducive to investigating different immunotherapeutic strategies based on melanoma antigen-specific and nonspecific immunostimulation or adoptive transfer of melanoma-specific activated T cells. However, the overall outcomes of immunotherapeutic clinical studies were not satisfactory [15, 16]. Melanoma can produce immunosuppressive cytokines, such as TGF-β, VEGF and IL-10, to create an immunosuppressive microenvironment that facilitates tumor growth and blocks antitumor immune responses [17].

Among immune suppressor cells, Treg cells and MDSCs are significantly increased in hosts with advanced malignancies. It is well established that Treg cells and MDSCs are essential for the control of autoimmune responses, and that their accumulations in tissues or peripheral blood of tumor-bearing mice are responsible for the suppression of anti-tumor immune effector T cell functions [18]. Cancer cells can modulate anti-tumor immune responses indirectly through the activation of Treg cells and MDSCs [19]. It has been shown that the loss of regulatory function by depletion of tumor-induced Treg cells and MDSCs might enhance immune responses, resulting in tumor reduction, while the increased number of Treg cells and MDSCs effectively prevented tumor destruction [20].

In the present study, Notch1 expression in melanoma cells upregulated TGF-β1 mRNA expression and promoted TGF-β1 secretion in tumor microenvironment, which enhanced tumor-mediated inhibition of T cell proliferation and activation. Furthermore, Notch1 inhibited CD8^+^ cytotoxic T lymphocytes infiltration and IFN-γ release in tumor tissues. It could also up-regulate PD-1 expression on CD4^+^ and CD8^+^ T cells and increased the ratio of CD4^+^CD25^+^FoxP3^+^ Treg cells and CD11b^+^ MDSCs in tumor microenvironment. All these contributed to weakened anticancer immune responses and promoted tumor growth.

Interaction between Notch and TGF-β signaling has been identified in multiple tumors to regulate a wide variety of complicated tumor biological behaviors. For example, heightened Dll4/Notch signaling in tumor cells can magnify TGF-β-induced pSMAD2/3 signaling and is required to sustain TGF-β-induced tumor cell growth [21]. Moreover, inhibition of Notch signaling leads to attenuation of both basal and TGF-β1-induced TGF-β signaling in clear cell renal cell carcinoma cells, including an extensive set of genes known to be involved in migration and invasion [22]. Additionally, Notch signaling is required for the initiation of epithelial-mesenchymal transition and synergizes with TGF-β signaling pathways to promote the transition of endothelial cells to mesenchyme, as well as mesenchymal cell invasiveness [23]. Our current findings further identified that Notch activity was a contributor to elevating melanoma-derived TGF-β secretion, which led to the inhibition of immune cell proliferation and function, and blocked antitumor immune responses.

TGF-β is produced by tumor cells and tumor-infiltrating immune cells within the tumor microenvironment. The role of TGF-β in immune suppression includes the inhibition of T cell proliferation, cytokine production and cytotoxicity [24, 25], as well as the remodeling of the tumor microenvironment and the recruitment of immune suppressive cells [26, 27]. Under some conditions, TGF-β also inhibits antigen presenting cell function by suppressing cell maturation, inhibiting IFN-γ production and inducing major histocompatibility complex class II down-regulation [28, 29]. Our results showed low TGF-β1 secretion and rare infiltration of Treg cells and MDSCs in tumor microenvironment after Notch1 knocking down. Therefore, we speculated that reduction of Treg cells and MDSCs in tumor sites was a possible consequence of the down-regulation of tumor-derived TGF-β1 induced by Notch1 knockdown. In addition, there were more CD8^+^ cytotoxic T lymphocytes infiltration and higher IFN-γ release in tumor microenvironment, while tumor growth was significantly suppressed. This revealed a more powerful function of CD8^+^ cytotoxic T cells and more intense anticancer immune responses in vivo. The opposite effects were found in Notch1 overexpression cells. All these results strongly suggested that aberrantly elevated TGF-β1 secretion in response to abnormal activation of Notch signaling was a potential mechanism of melanoma-induced immunosuppression.

## Conclusions

In conclusion, we found that Notch1 signaling in melanoma cells facilitated tumor immune escape and promoted cancer progression via TGF-β1 secretion. Inhibition of Notch1 expression in melanoma cells might be considered as a potential therapeutic method for melanoma immunotherapy.
